# Supplementary material for: Development and Validation of a UPLC-MS/MS Method for the Quantification of Components in the Ancient Classical Chinese Medicine Formula of Guyinjian
Source: Molecules. 2022 Dec 6;27(23):8611. doi: 10.3390/molecules27238611 (PMC9738704; doi:10.3390/molecules27238611)
Supplement: Supplementary file 1 [file molecules-27-08611-s001.zip › molecules-2001900-supplementary.pdf]

Supplementary materials

Figure S1: Results of method specificity examination. Rehmannioside D (A), Morroniside (B), Loganin (C), Polygalaxanthone III (D), Liquiritin (E), Hyperoside (F), Verbascoside (G), 3',6-Disinapoylsucrose (H), Ginsenoside Re (I), Ginsenoside Rg1 (J), Ginsenoside Rb1 (K), Tenuifolin (L), Glycyrrhizic acid (M), Schisandrin (N).

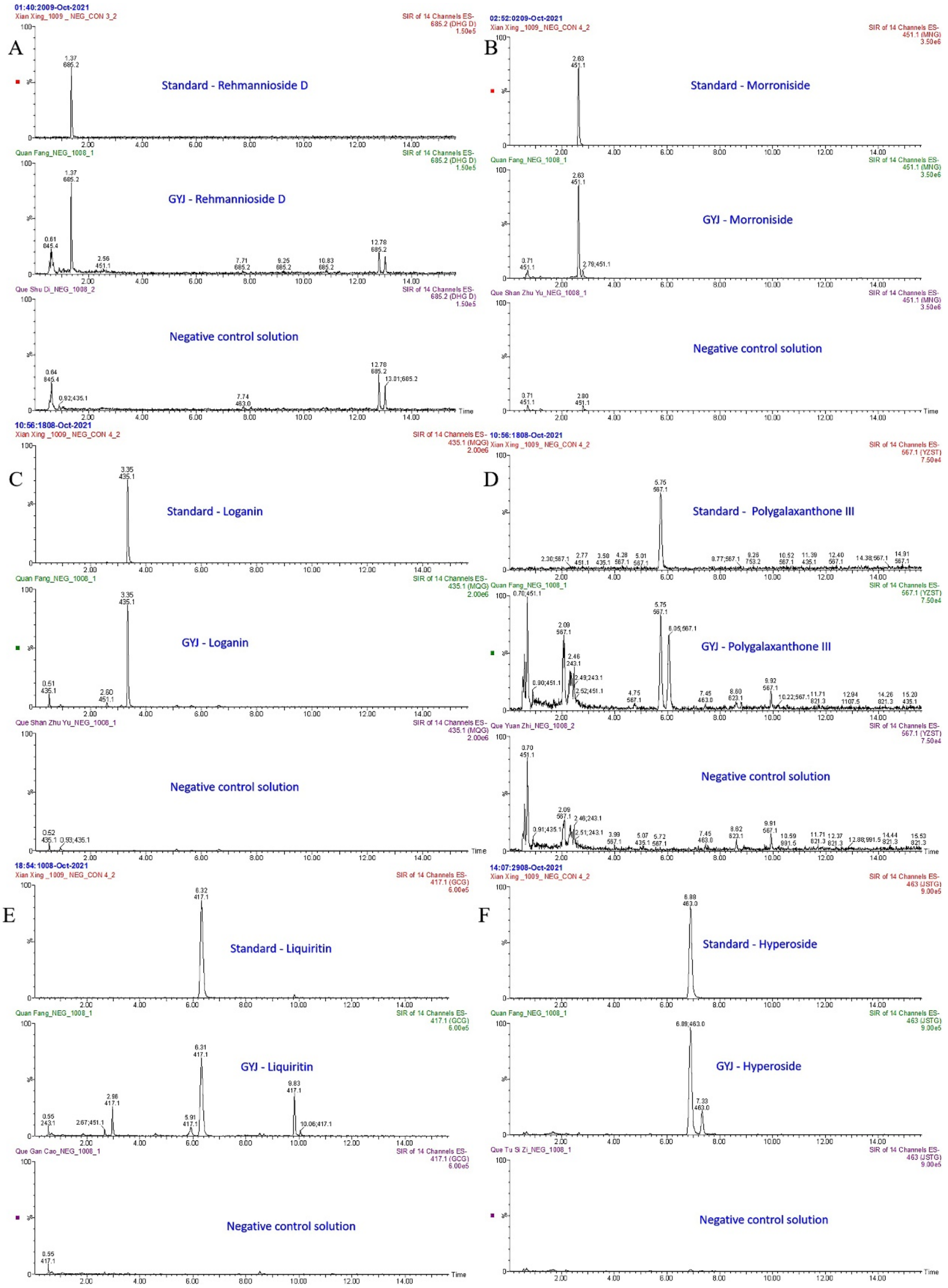

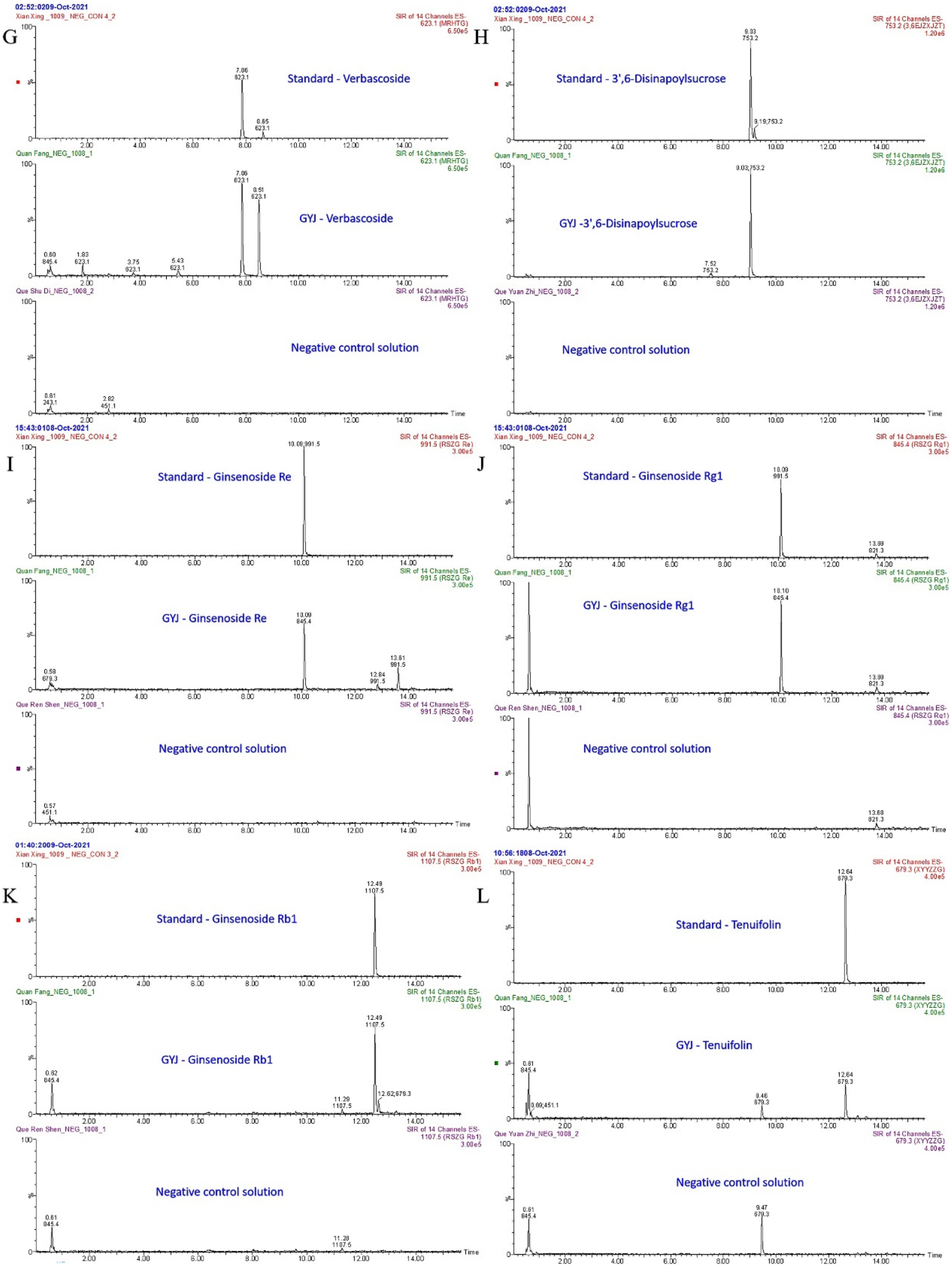
G
Standard - Verbascoside
GYJ - Verbascoside
Negative control solution
H
Standard - 3',6-Disinapoylsucrose
GYJ -3',6-Disinapoylsucrose
Negative control solution
I
Standard - Ginsenoside Re
GYJ - Ginsenoside Re
Negative control solution
J
Standard - Ginsenoside Rg1
GYJ - Ginsenoside Rg1
Negative control solution
K
Standard - Ginsenoside Rb1
GYJ - Ginsenoside Rb1
Negative control solution
L
Standard - Tenuifolin
GYJ - Tenuifolin
Negative control solution

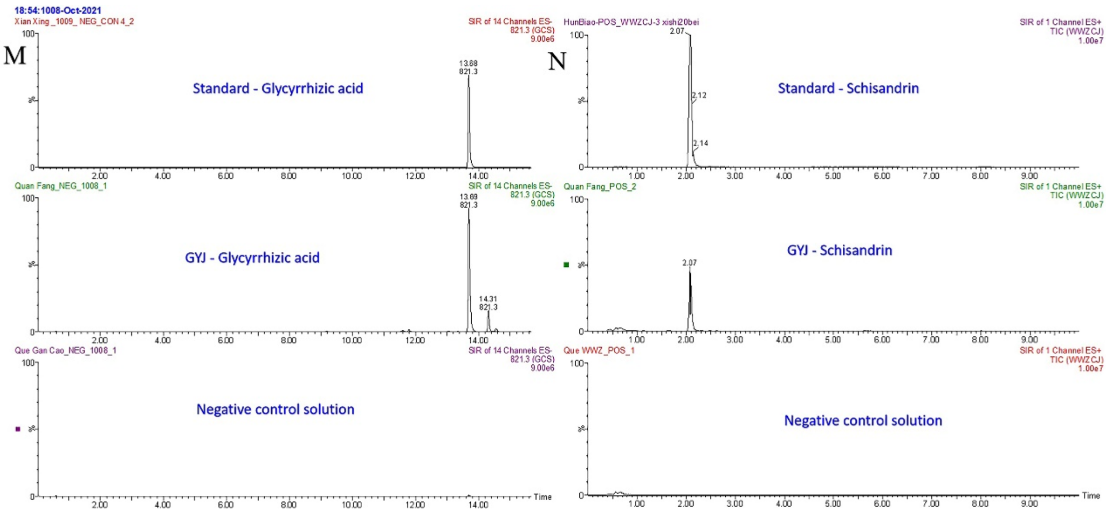
M
N
18:54:1008-Oct-2021
Xian Xing_1009_NEG_CON 4_2
SIR of 14 Channels ES-
821.3 (GCS)
9.00e6
Standard - Glycyrrhizic acid
13.88
821.3
Quan Fang_NEG_1008_1
GYJ - Glycyrrhizic acid
13.69
821.3
14.31
821.3
Que Gan Cao_NEG_1008_1
Negative control solution
HunBiao-POS_WWZCJ-3 xishi20bei
SIR of 1 Channel ES+
TIC (WWZCJ)
1.00e7
Standard - Schisandrin
2.07
2.12
2.14
Quan Fang_POS_2
GYJ - Schisandrin
2.07
Que WWZ_POS_1
Negative control solution
Time

Table S1: Information of standard products.

Compound	Molecular Formula	Molecular Weight	Purity	Supplier	Batch number	CAS
Rehmannioside D	$C_{27}H_{42}O_{20}$	686.610	98.30%	Chengdu Purfield Biotechnology Co., Ltd.	20050703	81720-08-3
Morroniside	$C_{17}H_{26}O_{11}$	406.382	HPLC＞98%	Chengdu Refensi Biotechnology Co., Ltd.	M-027-161226	25406-64-8
Loganin	$C_{17}H_{26}O_{10}$	390.382	99.20%	National Institutes for Food and Drug Control	111640-201707	18524-94-2
Polygalaxanthone III	$C_{25}H_{28}O_{15}$	568.481	96.50%	National Institutes for Food and Drug Control	111850-201705	162857-78-5
Liquiritin	$C_{21}H_{22}O_{9}$	418.39	95.00%	National Institutes for Food and Drug Control	111610-201908	551-15-5
Hyperoside	$C_{21}H_{20}O_{12}$	464.376	94.90%	National Institutes for Food and Drug Control	111521-201809	482-36-0
Verbascoside	$C_{29}H_{36}O_{15}$	624.587	HPLC＞98%	Chengdu Refensi Biotechnology Co., Ltd.	M-011-170629	61276-17-3
3',6-Disinapoylsucrose	$C_{34}H_{42}O_{19}$	754.686	HPLC＞95%	Chengdu Refensi Biotechnology Co., Ltd.	F-013-170426	139891-98-8
Ginsenoside Re	$C_{48}H_{82}O_{18}$	947.154	93.40%	National Institutes for Food and Drug Control	110754-201827	51542-56-4
Ginsenoside Rg1	$C_{42}H_{72}O_{14}$	801.013	92.40%	National Institutes for Food and Drug Control	110703-201832	22427-39-0
Ginsenoside Rb1	$C_{54}H_{92}O_{23}$	1109.295	91.20%	National Institutes for Food and Drug Control	110704-201827	41753-43-9
Tenuifolin	$C_{36}H_{56}O_{12}$	680.823	91.60%	National Institutes for Food and Drug Control	111849-201705	20183-47-5
Glycyrrhizic acid	$C_{42}H_{62}O_{16}$	822.932	HPLC＞98%	Sichuan Vikki Biotechnology Co., Ltd.	201005	1405-86-3
Schisandrin	$C_{24}H_{32}O_{7}$	432.507	99.90%	National Institutes for Food and Drug Control	110857-201714	7432-28-2
