# Supplementary figures and images for: Development and Validation of a UPLC-MS/MS Method for the Quantification of Components in the Ancient Classical Chinese Medicine Formula of Guyinjian
Source: Molecules. 2022 Dec 6;27(23):8611. doi: 10.3390/molecules27238611 (PMC9738704; doi:10.3390/molecules27238611)

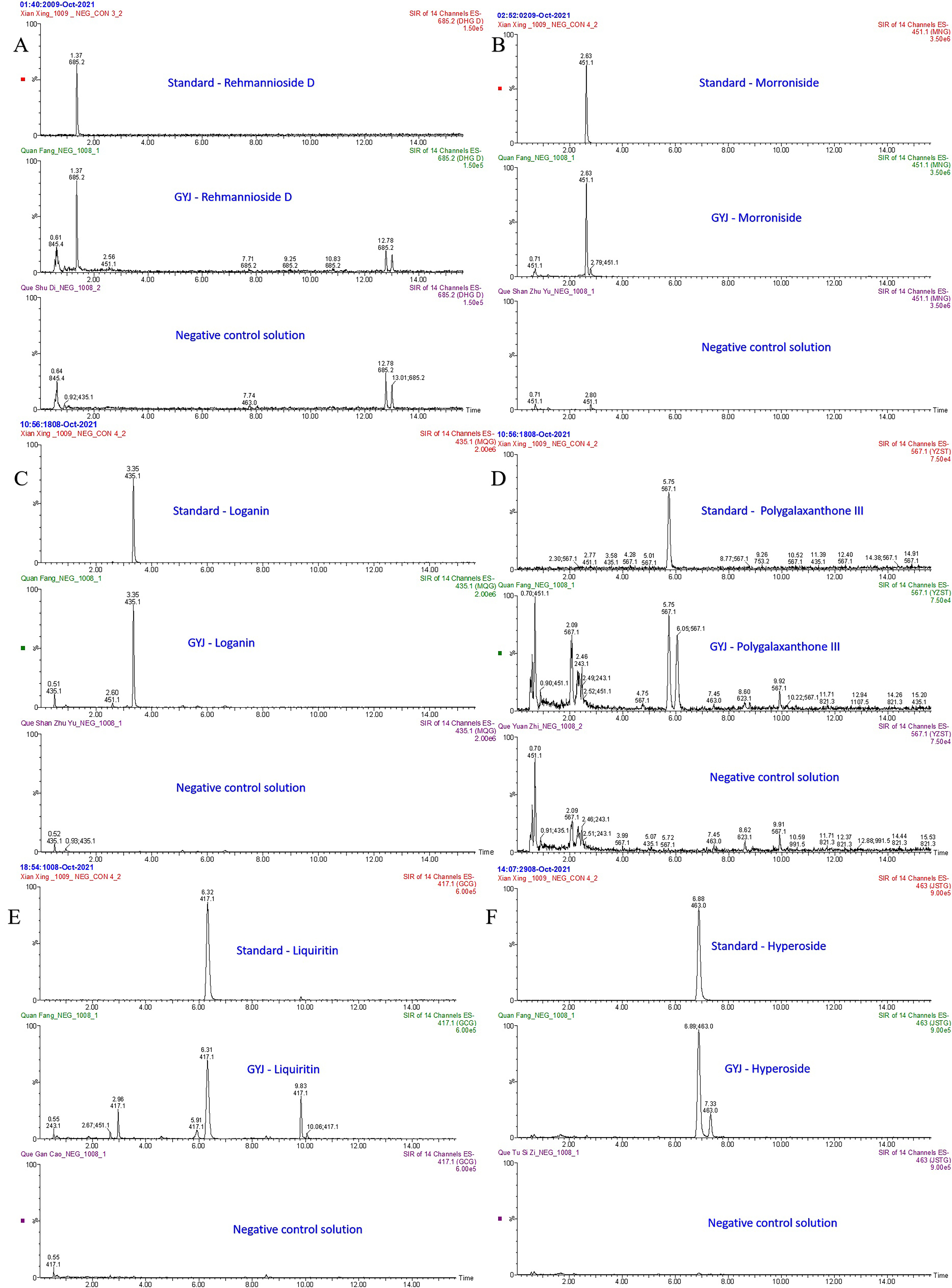

Supplement: Supplementary file 1 [file molecules-27-08611-s001.zip › S1-6.jpg]

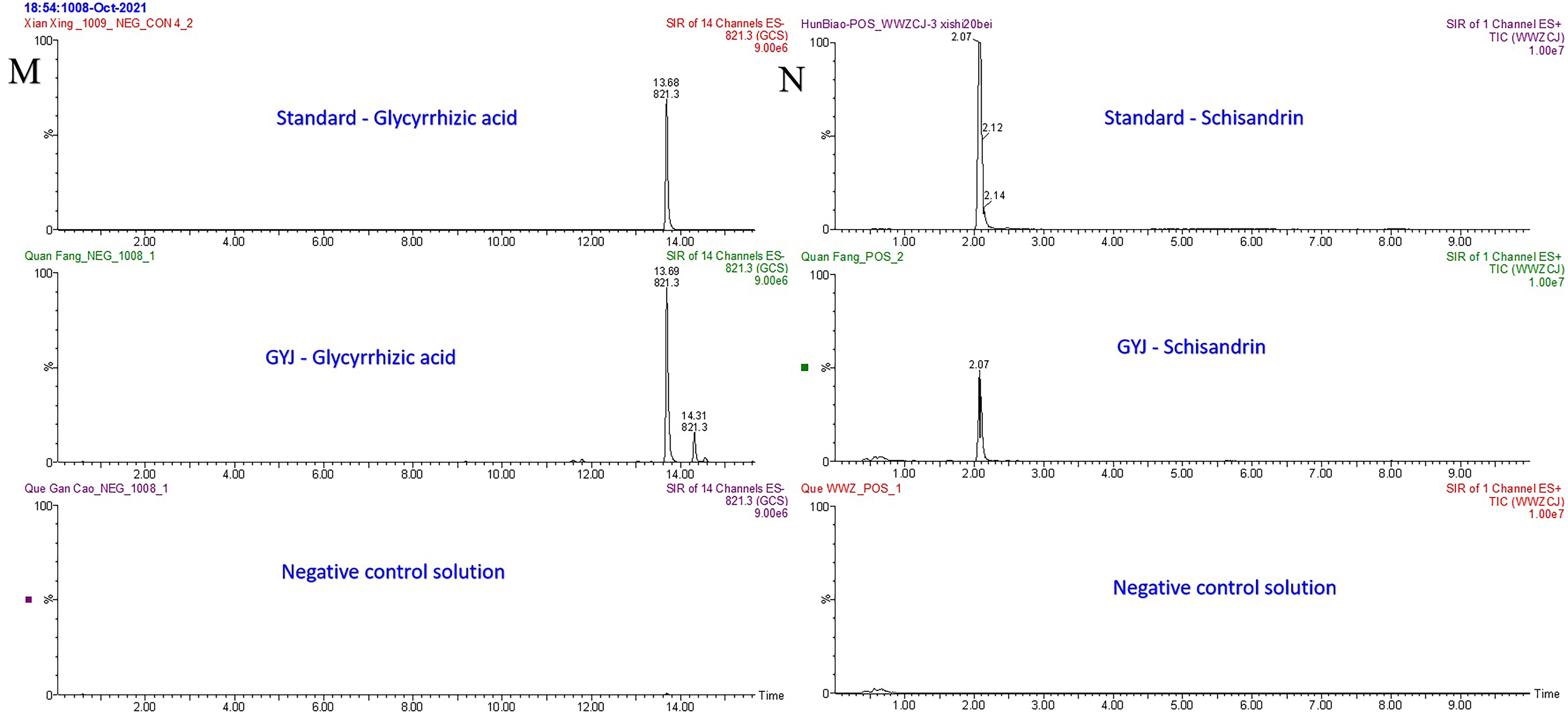

Supplement: Supplementary file 1 [file molecules-27-08611-s001.zip › S13-14.jpg]

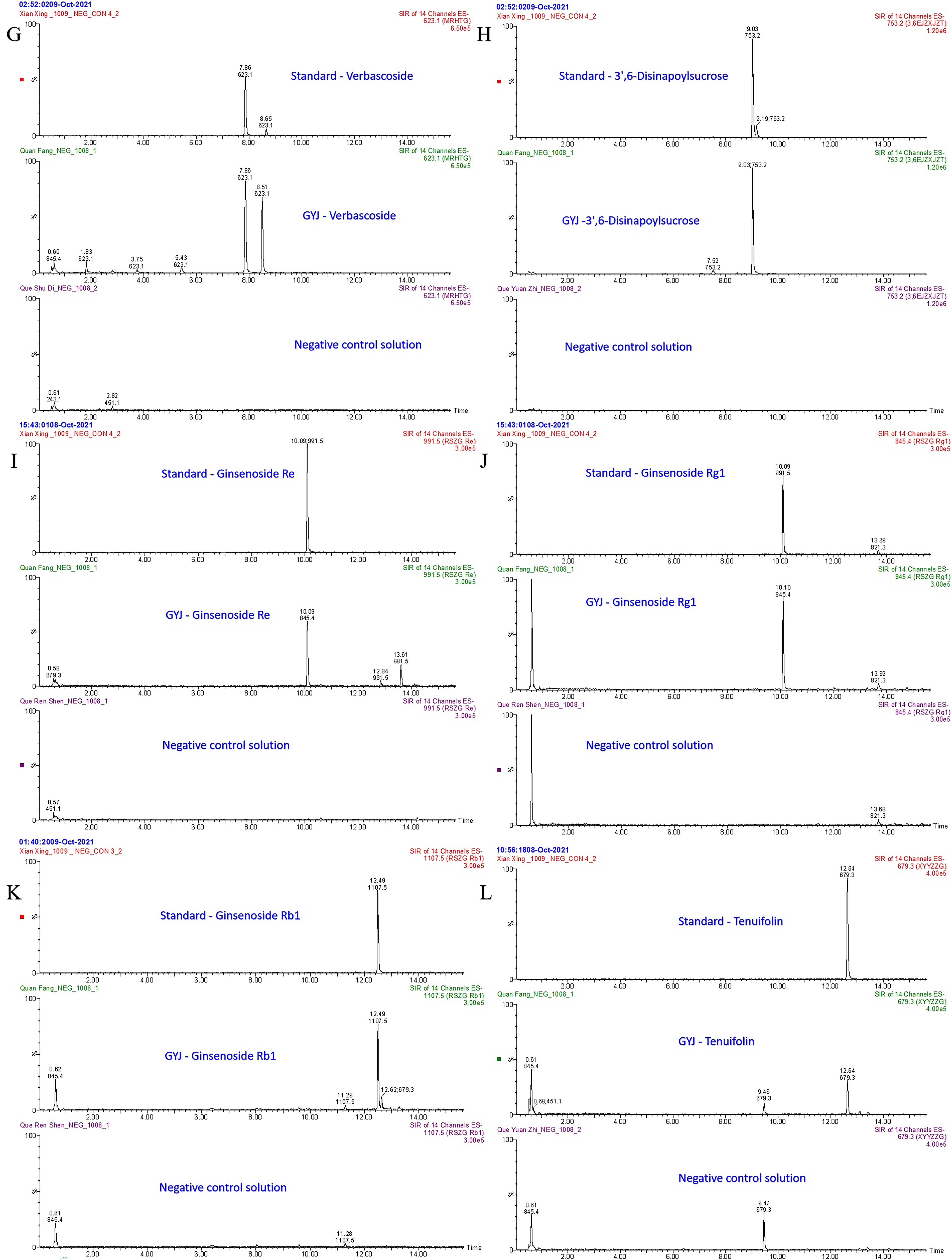

Supplement: Supplementary file 1 [file molecules-27-08611-s001.zip › S7-12.jpg]
